# Baseline Perceptions of Women With Gestational Diabetes Mellitus and Health Care Professionals About Digital Gestational Diabetes Mellitus Self-Management Health Care Technologies: Interview Study Among Patients and Health Care Professionals

**DOI:** 10.2196/51691

**Published:** 2023-12-19

**Authors:** Ladan Safiee, Daniel Rough, Priya George, Roselyn Mudenha

**Affiliations:** 1 School of Science and Engineering University of Dundee Dundee United Kingdom; 2 Ninewells Hospital NHS Tayside Dundee United Kingdom

**Keywords:** gestational diabetes, self-management, mobile health, mHealth, qualitative study, mobile phone

## Abstract

**Background:**

Gestational diabetes mellitus (GDM) is a significant medical complication of pregnancy that requires close monitoring by a multidisciplinary health care team. The growing sophistication of mobile health (mHealth) technology could play a significant supporting role for women with GDM and health professionals (HPs) regarding GDM management.

**Objective:**

This study included 2 phases. The aim of phase 1 was to explore the perceptions of HPs and women with GDM regarding the use of mHealth for GDM self-management and to identify their needs from these technologies. The aim of phase 2 was to explore the perceptions of women with GDM about their experiences with a state-of-the-art app for managing GDM that was offered to them during the COVID-19 lockdown. This phase aimed to understand the impact that COVID-19 has had on women’s perceptions about using technology to manage their GDM. By combining both phases, the overall aim was to establish how perceptions about GDM self-management technology have changed owing to the pandemic restrictions and experience of using such technology.

**Methods:**

In total, 26 semistructured interviews were conducted in 2 phases. In phase 1, overall, 62% (16/26) of the participants, including 44% (7/16) of HPs, 50% (8/16) of women with GDM, and 6% (1/16) of women in the postpartum period with GDM history participated in the interviews. In phase 2, overall, 38% (10/26) of women with GDM participated in the interviews. NVivo (QSR International) was used to extract qualitative data, which were subjected to thematic analysis.

**Results:**

Phase 1 identified 3 themes from the interviews with women with GDM: fitting with women’s lifestyle constraints, technology’s design not meeting women’s needs, and optimizing the technology’s design to meet women’s needs. Overall, 3 themes were derived from the interviews with HPs: optimizing the technology’s design to improve the quality of care, technology to support women’s independence, and limitations in the care system and facilities. Analysis of phase-2 interviews identified 2 further themes: enhancing the information and functionalities and optimizing the interface design. In both phases, participants emphasized a simple and user-friendly interface design as the predominant positive influence on their use of technology for GDM management.

**Conclusions:**

The combined findings underlined similar points. Poor usability, data visualization limitations, lack of personalization, limited information, and lack of communication facilities were the prime issues of current GDM self-management mHealth technology that need to be addressed. The analysis also revealed how women with GDM should play a vital role in gathering the requirements for GDM self-management technology; some needs were identified from in-depth discussion with women with GDM that would be missed without their involvement.

## Introduction

### Background

Gestational diabetes mellitus (GDM) is defined as any degree of hyperglycemia with first recognition during pregnancy [1]. The prevalence of GDM in the United Kingdom is approximately 4% of all pregnancies [2]. However, the rate of GDM is likely to rise owing to population trends toward maternal obesity and advancing age of childbearing [3]. Various studies have shown GDM to be associated with serious maternal [4-6] and fetal complications [7-10]. Women with GDM are also at great risk of developing type 2 diabetes [11] and cardiometabolic disorders later in life [12]. Their infants are also more at risk of developing adulthood obesity and type 2 diabetes [7,13].

The aim of GDM management is to optimize maternal blood glucose (BG) levels through good control of diet, physical activity, and (in some cases) regular medication. Despite having support to manage GDM from health services, women with GDM encounter challenges and barriers that adversely affect the self-management process. Some examples of these challenges and barriers are lack of knowledge, lack of motivation [14], lack of appropriate recommendations based on patients’ values and beliefs, low level of family support, low self-efficacy [15], lack of knowledge about a diet plan [16], and lack of specific personal information [17]. Therefore, methods of optimizing glycemic control while reducing the GDM self-management challenges and the burden on women and services are needed. Recently, there is a tendency to empower women with GDM to have more control in the management of their condition by using technology that could shift the management of GDM from hospital-centered to community-centered and patient-centered care [18]. Technology could support women with GDM in optimizing their BG levels, thereby minimizing the adverse effect of GDM on both them and their babies. Furthermore, technology might be applied to address all the abovementioned barriers and offer advantages such as reducing patient traveling and waiting times [19], saving the time of medical practitioners [20], cost saving [21] for both the health care system and patients, improving convenience [22], and supporting community continuity of care.

With near-ubiquitous internet connectivity [23] and improving processing capabilities, smartphone apps are ideally placed to play an important role in the management of diabetes, particularly in improving patient lifestyle behavior, knowledge, attitude, and skills [24]. However, to provide mobile health (mHealth) care systems with acceptable quality, it is important to involve and engage users in the design procedure and development of these systems [25]. It is also important to identify their perceptions about the barriers to and advantages of using these systems [26].

Guidelines for women with GDM in the United Kingdom recommend reviews every 1 to 2 weeks at a hospital-based center by a multidisciplinary team from diabetes and antenatal care [27,28]. However, different parts of the United Kingdom follow different guidelines and care for GDM management [28,29].

### Study Aim

In recent years, state-of-the-art technology has evolved to provide a wide range of support to women with GDM in their self-management. Most of these innovative systems provide physiological support to women with GDM in monitoring their BG levels [20,30-33]. Monitoring blood pressure, ketonuria [20], and medication management [20,30,34] are among the other physiological features offered by some of these technologies. They also provide lifestyle support to women with GDM, such as managing or monitoring diet [20,31-33] or physical activity [20,31-33]. Information support is another feature in some systems to help women understand GDM and optimize their self-management [30,32,33]. In addition, a communication feature provides support from health professionals (HPs) to women with GDM [30,33]. Previous studies have explored the experiences of women with GDM or HPs with current mHealth technologies, including the adoption of or perceptions about specific mHealth apps such as Pregnant+ [35,36], my Diabby [37], and TeleGDM [26] and about the general use of mHealth during pregnancy [38]. In this study, we build on these existing studies by adding novel knowledge about the role of women with GDM in identifying the requirements for a GDM self-management system. Furthermore, we explore how the current state-of-the-art technology meets women’s needs regarding GDM self-management.

This study included 2 phases. Phase 1 was conducted before the COVID-19 pandemic, at which time, all participants were using paper logbooks. This phase aimed to explore and examine the perceptions of women with GDM and HPs about how technology could support women to meet their GDM self-management needs. Phase 2 was conducted in 2022 (following cessation of the legally enforced restrictions) and aimed to explore the perceptions of women with GDM about their experience of using a specific GDM self-management app called *GDm-Health* that was offered to them after COVID-19 restrictions had been relaxed. This second phase enabled us to understand how well a state-of-the-art GDM self-management technology in the United Kingdom [30] addressed women’s needs that were identified in phase 1. Furthermore, it provided insights into how using specific mHealth technology may affect the way women view such support for managing their GDM condition.

## Methods

### Study Design

A qualitative study was conducted in the Tayside region of Scotland. Data collection occurred in 2 phases. The first phase was from November 2019 to March 2020, which consisted of semistructured interviews. It explored the perceptions of women with GDM, women in the postpartum period who have had GDM, and HPs about using technology to support women with GDM self-management. In the second phase, semistructured interviews were conducted from April 2022 to December 2022 to discuss the experiences of women with GDM regarding their use of the GDm-Health app.

### Recruitment

Recruitment was conducted at the antenatal clinic in Ninewells Hospital, Dundee, a large teaching hospital in the Tayside region of Scotland, which runs a weekly GDM clinic. Women with GDM were identified by HPs at the antenatal clinic. An information sheet and a reply form were given to potential participants. The first author was also available at the GDM clinic to explain the study to women with GDM or answer any of their questions.

Furthermore, the Hospital’s Women and Baby Facebook group was used to recruit women in the postpartum period who have had GDM.

Recruitment inclusion criteria for women were to be aged ≥18 years, able to consent, and either diagnosed with GDM and a minimum gestational age of 16 weeks or in the postpartum period within 5 years of a GDM diagnosis with a healthy baby. HPs were eligible for recruitment if they worked with women with GDM or diabetes. Participants were excluded if they did not speak or understand English, had significant communication difficulties, or had preexisting diabetes (type 1 and type 2). In addition, 10 women with GDM were interviewed between April 2022 and December 2022 to gather their perceptions about the GDm-Health app that had been offered to them. There was no minimum use time of the app for recruitment.

### Interviews

Phase-1 interviews were primarily conducted at the hospital where the recruitment occurred. Semistructured interviews were conducted with 16 participants comprising 8 (50%) women with GDM, 1 (6%) woman in the postpartum period with a history of GDM, and 7 (44%) HPs who worked with patients with GDM. The interviews were conducted from November 2019 to March 2020. Participants were interviewed in a private room in the antenatal clinic or Strathmore Diabetes Centre at Ninewells Hospital. Women with GDM were interviewed before or after their appointments, and HPs were interviewed in their free time during working hours (between appointments) or after their work. Interviews consisted of 2 sections. The first section gathered interviewees’ perceptions about digital health care technologies for GDM self-management. The second section explored attitudes toward the involvement of women with GDM in the design stage of these technologies and the design process. This paper only includes the first part of the interviews of phase 1. The first section of interviews lasted an average of 30 (SD 12.45) minutes for women, depending on their conditions and availability, and an average of 22 (SD 5.56) minutes for HPs. To ensure that the interviews followed a similar structure, an interview guide (Multimedia Appendices 1 and 2) was used as an aid during the sessions. The interview guide was developed for the purpose of understanding participants’ perceptions in 2 areas. First, we sought participants’ perceptions about GDM, its management, and current care limitations and problems regarding GDM management. Second, we sought participants’ opinions about using technology, its benefits and drawbacks, and their needs from technology to help them manage their GDM. Furthermore, we were also interested in participants’ opinions about their confidence and comfort in receiving care remotely in comparison with clinical visits.

In phase 2, semistructured interviews were conducted with 10 women with GDM through Teams (Microsoft Corporation). These interviews also contained 2 parts: the first part involved participants testing the proposed paper prototype, and the second part focused on participants’ experiences with GDm-Health. This paper only discusses the second part of the interviews, which lasted between 10 and 20 minutes (the interview guide for phase 2 is available in Multimedia Appendix 1). The interview guide for phase 2 was developed to understand women’s opinions about using state-of-the-art GDM self-management technology and how (or if) it met their needs by exploring the benefits and limitations.

### Analysis

Thematic analysis with an inductive approach was used to develop themes from interview data following the 6 steps outlined by Braun and Clarke [39].

After becoming familiar with the data by reading the interview transcripts multiple times, relevant data for our study’s aims were identified. Next, codes were identified for each segment of the data. Segments of data associated with each code were reviewed iteratively by the first and second authors to ensure a shared understanding. During this process, some codes were merged, deleted, or broken into new codes. Then, all relevant codes were combined and sorted into potential themes or subthemes. These were reviewed and refined iteratively to reflect our study’s aims. Identification of initial themes was conducted by the first author. Refinement was conducted through Level One (reviewing the codes of each theme to identify coherent patterns) and Level Two analysis (reviewing the themes to assess whether they reflect the entire data set) by the first and second author. Interrater reliability was not assessed, consistent with the process recommended by Braun and Clarke [39].

### Ethical Considerations

The study protocol for phase-1 interviews was approved by the West of Scotland Research Ethics Committee in September 2019 and from Research and Development National Health Service (NHS) Tayside in October 2019. The modified study protocol for phase-2 interviews was approved by West of Scotland Research Ethics Committee in December 2021 and Research and Development in NHS Tayside in January 2022 (Integrated Research Approval System ID 240156; Research Ethics Committee reference number 19/WS/0134; Tayside reference number 2019DM02). Women with GDM were offered Amazon vouchers worth £15 (US $18.86) as compensation for their time spent in both phases.

## Results

### Phase-1 Results (Women With GDM)

#### Overview

Women’s average age was 31 (SD 5.052) years. The average gestational age was 31 (SD 4.413) weeks for 78% (7/9) of the participants. One participant was in the postpartum period, and another participant’s gestational age was missing. Among different devices, all women (9/9, 100%) were using smartphones on a daily basis (Multimedia Appendix 3).

In phase 1, women with GDM, women in the postpartum period with a history of GDM, and HPs provided their perceptions about health care technologies to support GDM self-management. The views of women and HPs are reported separately throughout the analysis. A summary of themes and subthemes for women’s perceptions in phase 1 is shown in Table 1. Definitions of the themes can be found in Multimedia Appendix 4.

**Table 1:** Summary of themes and subthemes obtained from women’s perceptions in phase 1.

Themes and subthemes			Key findings
**Fitting with women’s lifestyle constraints**			
	N/A^a^	Reducing the need for travel to the clinic Reducing the need for personal arrangements Saving time and personal costs Pervasiveness of smartphones	
**Technology’s design not meeting women’s needs**			
	Need for well-being support	Need for support from technology to change lifestyle behavior Lack of physical and emotional support through technology	
	Data integrity concerns	Concerns about security and data privacy Concern about the accuracy of the reported data	
	Inadequate information for women’s needs	Inadequate and overwhelming information Lack of personalized information Reliability concerns about technology’s information	
**Optimizing the technology’s design to meet women’s needs**			
	Data recording options	Need for a place to record blood glucose levels, food, and physical activity	
	Empowerment through understanding	Need for different ways to visualize data Access to all data in a single place Access to data analysis	
	Improving communication	Need to share data with HPs^b^ Different communication channels with HPs	
	Optimizing the user interface design	Need for user-friendliness and simplicity Intuitive categorization of options	

^a^N/A: not applicable.

^b^HP: health professional.

#### Theme 1: Fitting With Women’s Lifestyle Constraints

Technology that can be integrated into a busy lifestyle was of primary importance for women with GDM. Using GDM self-management technology was seen to potentially assist with their busy lives by reducing travel to in-person appointments, reducing personal arrangements (eg, childcare), and saving time and costs associated with these. Although participants recognized the benefits of in-person appointments, they were clear about the impact that attending these appointments has on their well-being in terms of stress, energy, and inconvenience:

I drive in order to get here [the GDM clinic] normally for 40 minutes but today it took an hour and whatever because of wind and traffic.

Woman 6

The pervasiveness of smartphones was also acknowledged as facilitating the adoption of mHealth technology. Participants suggested how it could eliminate carrying additional paper documents or equipment and believed that a smartphone app would be more comfortable than using a logbook:

Just always [have] my phone on me, so as I was saying, having to carry things round, whereas I always [have] my phone and I would probably as I ate something, would put it in immediately, and be able to sort of have it there.

Woman 4

However, women acknowledged that the pervasiveness of technology did not guarantee its convenience. Technical problems with apps, problems with accessing the internet, and problems with finding a suitable environment for web-based visits were raised as limitations of app-based self-management:

...Not everybody has the option of being able to move themselves away into a private area or whatever, if they don’t have regular access to the internet.

Woman 9

#### Theme 2: Technology’s Design Not Meeting Women’s Needs

##### Need for Well-Being Support

All women (9/9, 100%) believed the lack of physical or emotional support from HPs to be a primary concern of using technology-based self-management. They perceived that if care was completely provided through remote technology, this would not address some of their well-being needs, such as the need to be examined by HPs or building proximity and trust with their HPs:

The midwives do such a physical exam as well, I think, that would maybe concern me if someone was only offering me the remote monitoring.

Woman 4

Women also believed that a lack of emotional support could have a significant impact on single women with no support or on women with “mental illness” (woman 7). Thus, they emphasized the importance of face-to-face clinical appointments as a primary means of care for women with GDM, with technology acting as a complementary addition:

That if it went totally remote some people might not, they might feel alienated if they’ve not got a support network, they might, um, might have anxiety so you know, actually coming out might be good for them.

Woman 8

However, they also found it challenging to significantly change their lifestyle behavior and were overwhelmed with the initial information they received about managing their GDM. Women thought that technology could provide support to cope with these initial challenges of changing their lifestyle and managing their condition:

*I mean I would have died for a little app.... Just something simple, just going on to it, going right okay, “oh I wonder if I can have this snack”or...write “my bloods were so and so, I’ll just pop in here.”* [Woman 2]

##### Data Integrity Concerns

Security and data privacy were significant concerns of 33% (3/9) of the participants, who were uncertain how their data would be “transferred from phone over to the NHS or to the doctors” (woman 6). They also expressed concern over whether their data would be stored “securely or privately” (woman 6) and their “confidence in the organization” (woman 6) responsible for the process. Woman 3 was also concerned about the impact of a data breach on the system:

What if that system was hacked, like there’s so many things that can go wrong with these systems.

Woman 3

Moreover, woman 8 doubted the accuracy of data that women would report. She indicated a possibility of not adopting GDM self-management correctly while reporting the wrong data to avoid attending face-to-face GDM clinical appointments:

...But somebody might just put them all like really good results because they can’t be bothered coming in to visit.

Woman 8

##### Inadequate Information for Women’s Needs

Requirements for a GDM self-management app include the presentation of relevant information, which was a prominent issue in women’s discussion about their needs. Women mentioned inadequate information, overwhelming information, lack of personalized information, and poor navigability as issues with relevant websites:

Like the NHS one [website], I didn’t think gave you enough information on gestational diabetes itself. It was mainly type 1 and type 2.

Woman 4

Whereas the Diabetes UK I do find overwhelming.

Woman 5

[An app] *has to be personalized..., but it has to be a specific, something that’s really, really useful, otherwise, it’s just another app.* [Woman 7]

Irrespective of design, women were concerned about the reliability of the information provided on both bespoke websites and social media groups. Women emphasized the need for a trusted source after having found disparate or even contradictory advice about GDM management:

I think it’s hard to find reliable information yourself and reliable sites because anybody could be writing these things.

Woman 1

Generally, women emphasized advocating technology as complementary care for the standard care owing to its limitations in addressing some women’s needs.

#### Theme 3: Optimizing the Technology’s Design to Meet Women’s Needs

##### Data Recording Options

Women outlined some important elements for optimizing the usefulness of technology for supporting their GDM self-management. Mainly, they indicated that the ability to record BG levels, food, physical activity such as step count, and other comments would be helpful for GDM management:

The recording obviously of your food diaries and your blood sugars and perhaps being able to record the trends somehow.

Woman 1

Most women (6/9, 67%) agreed that technology would support self-management by improving logging of information, such as an “automatic space and place to enter everything that [women] would need” (woman 6). In addition, women also valued the ability to connect the app with other technology to transfer data automatically between them:

...Also you know if it could tie it to the likes of your Fitbit or something, you know or your, your smartwatch.

Woman 8

##### Empowerment Through Understanding

Improving the presentation of data by providing “graphs” (woman 9), “videos, 3D demonstrations” (woman 5), and other data in a single place would help women to understand their condition “much more in depth” (woman 6) and increase their self-empowerment in managing their GDM condition. Women perceived that technology could provide additional information to “analyze your own data” (woman 1), including summaries, averages, and means of identifying correlations in their data to visualize how variables influence each other:

It’d be quite interesting to see actually that day you did 10000 steps, and that was the impact or not. Yeah I think that would be quite good.

Woman 8

All women (9/9, 100%) also emphasized the importance of accessing GDM information, particularly after diagnosis. They believed that technology could provide instant access to a vast scope of information to support a better understanding of their GDM condition and its self-management and give women reassurance and encouragement to move forward:

...That’s what’s going to want me to go on to the app and move me forward, but more importantly that’s what’s going to give me the knowledge as a patient to be able to help myself and give the reassurance that I need.

Woman 5

Despite women appreciating the care received from the NHS, woman 2 indicated that she was overwhelmed with the amount of verbal information received at the introductory meeting organized with NHS staff. Furthermore, woman 7 indicated that it would be better if the information was personalized at the meeting based on their backgrounds and knowledge. Women also mentioned receiving leaflets from HPs, but woman 2 found these to be inconvenient and found their information to be insufficient. However, they valued having something such as an app to remind themselves about important information:

...Honestly it’s a lot of information to take in and sometimes you don’t take it in, even somewhere to refer back to and go “ah, that’s what they were on about.”

Woman 2

##### Improving Communication

Many women (6/9, 67%) saw having different means of communication with HPs via technology as useful in supporting their GDM self-management. For example, women emphasized the value of real-time care and sharing data with HPs through technology for optimizing their GDM management:

If you noticed sort of your blood sugar going up, then, I guess if there was an app, that they could see your records, then you could see while they were on the phone with you.

Woman 4

Despite a few women being uncertain about the reliability of the information on social media, most (7/9, 78%) were interested in having a “forum” (woman 9), “message boards” (woman 6), or “a chat function” (woman 1) to interact with other women with GDM:

Maybe experiences from people who have gone through it in your area so they’re local to you, um, so kind of a chat function.

Woman 1

##### Optimizing the User Interface Design

Most women (6/9, 67%) emphasized that the interface design of technology would influence its use. For example, woman 5 found herself overwhelmed with the information in Diabetes UK and found it poorly designed for finding information:

...The Diabetes UK I do find overwhelming. There’s so much information, and it doesn’t seem to me to be bookmarked or, or in any particular order when you get on to it.

Woman 5

They indicated that, in contrast to Diabetes UK, an app’s interface design should be “user-friendly” (woman 1), “simple” (woman 6), “easy to use” (women 6 and 8), and “very straightforward” (woman 8) and provide “well-categorized information” (woman 5):

Em, like I’ve said if the app was complicated to use, it was a bit time consuming a bit of a faff.

Woman 8

### Phase-1 Results (HPs)

#### Overview

In total, 7 HPs provided their perceptions about health care technologies to support GDM self-management. HPs’ average age was 40 (SD 8.802) years. Of the 7 HPs, 2 (29%) were employed as dietitians, 2 (29%) as diabetes specialist nurses, 2 (29%) as consultants, and 1 (14%) as a midwife. All HPs (7/7, 100%) used smartphones daily for different tasks and different situations (Multimedia Appendix 5).

In general, HPs believed that technology could play an important role in GDM management, and all (7/7, 100%) felt that the convenience and pervasiveness of technology would be impactful factors for using technology over traditional care. A summary of themes and subthemes for HPs’ perceptions in phase 1 is shown in Table 2. Full definitions of the themes are available in Multimedia Appendix 4.

**Table 2:** Summary of themes and subthemes obtained from the perceptions of health professionals (HPs) in phase 1.

Themes and subthemes			Key findings
**Optimizing the technology’s design to improve the quality of care**			
	Optimizing the efficiency of care and communication	Supporting HPs in making medical management decisions Updating women’s medical care quickly	
	Decreasing HPs’ workload and improving women’s well-being	Saving time for HPs Reducing clinical appointments	
**Technology to support women’s independence**			
	Helping women to understand their data	Visualizing data in different ways (eg, charting and color coding) All data in a single place Correlations between data streams	
	Increasing women’s knowledge and motivation	Direct access to information Provision of information in different formats for people with various learning abilities	
	User interface design	Need for usability and intuitiveness More interactivity Simplicity of data visualizations	
**Limitations in the care system and facilities**			
	N/A^a^	Reliability concerns such as hacking Technical problems, such as failure of the system Lack of in-person assessment; fetus safety concern	

^a^N/A: not applicable.

#### Theme 1: Optimizing the Technology’s Design to Improve the Quality of Care

##### Optimizing the Efficiency of Care and Communication

All HPs (7/7, 100%) expressed that improving technology design for remotely monitoring and communicating with women would optimize the efficiency of care and quality of GDM management. Furthermore, technology would support HPs in making medical management decisions and facilitate the communication between women and HPs, such as updating women’s medical treatment without having to go to a health care center:

...The patient can phone and say “my sugars have been bla, bla” and I can say, what’s your name? And I can actually go and look at it, so you know, you’ve got instant access to things.

HP2

However, most HPs (4/7, 57%) emphasized the design of technology as an important factor that would influence the efficiency of their work. They perceived the need for “a good format” (HP1) and an “easy” (HP2) and “quick” (HP3) layout that avoids “multiple screens” (HP1) to enhance the use of technology and the efficiency of their work regarding GDM management.

##### Decreasing HPs’ Workload and Improving Women’s Well-Being

Most HPs (5/7, 71%) believed that, in addition to the convenience of using technology for women with GDM, saving time and decreasing their workload and clinical appointments would influence their work positively. They believed that suitable technology would help manage women with GDM, particularly with increasing population trends in the prevalence of GDM and limitations in NHS diabetes resources:

We can still have, em, contact, get the information we need from them but reduce their clinic visits, and then obviously our workload as well.

HP4

Overall, HPs valued using technology from different perspectives for improving the quality of care and women’s lives. However, they emphasized ease of use as an important aspect of technology that could affect the efficiency of HPs’ work.

#### Theme 2: Technology to Support Women’s Independence

##### Helping Women to Understand Their Data

All HPs (7/7, 100%) perceived that technology could help women to record their data and understand their data through different data visualizations (eg, color-coded charts) in a single place and find correlations between data streams. These could then lead to optimizing their independence, stimulate them to monitor their GDM condition, and support their lifestyle modification:

If something could give women a graph representation which actually gives them even colour coding that would be amazing because it would help women to recognise when the sugars are up.

HP3

##### Increasing Women’s Knowledge and Motivation

HPs believed that constant access to information was another useful factor of technology that could result in enhancing women’s independence. HPs appreciated women having direct access to information such as food (particularly recipes) or exchanging their experiences. Furthermore, HP7 stated that technology could help people with different learning abilities and lifestyle conditions by providing information in various formats:

Now some people have very busy lifestyles or have the inability to read, therefore, it [Gestational Diabetes UK] uses videos on the website.

HP7

However, some HPs were also concerned about huge limitations regarding the availability of GDM management information and the reliability and accuracy of web-based information:

...Because obviously patients can go off Googling and get lost in all sorts of places and we don’t know that the advice that they’re reading is necessarily backed up by any sort of evidence.

HP5

##### User Interface Design

HPs suggested that technology should be* *“user-friendly, intuitive” (HP1)*,* “easy, fast*,* and more interactive” (HP2), “with simple data visualizations” to help women understand data, for example, “using color coding” (HP3) to easily identify hyperglycemia or hypoglycemia values in their data. Some HPs believed ease of use to be the most important factor, owing to variation in the intellectual levels among women with GDM:

What we should be providing is something easy enough for patients at that intellectual ability to understand easily and not at the level of obviously somebody who’s got a degree.

HP3

However, HPs expressed their concerns about the layout of existing information sources such as Diabetes UK for being overwhelming, not specific to GDM, and difficult for finding GDM information:

What I don’t like is that [Diabetes UK] is a hectic website, so for people to actually go and find things, it’s not as easy.

HP2

Generally, HPs emphasized the usability and interface design of technology as significant factors.

#### Theme 3: Limitations in the Care System and Facilities

HPs expressed the limitations of existing GDM management technology in the care system as an important factor preventing the full adoption of technology for GDM management. They indicated that a lack of Bluetooth in BG meters was a problem for the automatic transmission of BG readings to other devices. Although they could download the BG reading from the glucose meter to their computer, this process is time consuming and “lengthy” (HP7) in busy clinics:

...At the moment we don’t have a meter that would connect remotely...the meters that we gave patients, they can’t remotely connect that so that we can access it.

HP1

In contrast to the ideals of convenience, HPs discussed the inconvenience of using technology owing to its reliability issues and “relying on the patient having the technology” (HP7). Similar to women with GDM, they also expressed reliability concerns such as “hacking and security of the system” (HP3)*,* technical problems such as “failure of the system” (HP1 and HP3)*,* “viruses” (HP2), and incompatibility between different devices or systems.

Lack of in-person assessment, either emotional or physical, was another prime limitation of using technology that was discussed by HPs:

If they came in I would maybe be able to pick up “oh I know this woman,” if I think, “oh, she doesn’t seem herself,” there’s maybe something wrong, but you can’t see that through it [technology].

HP4

They also supported their concern by explaining that diabetes was not the only aspect of managing women with GDM; progress of their pregnancy also required physical examination to assure the safety of the fetus.

Finally, 43% (3/7) of the HPs discussed the necessity of equality in providing care for women with GDM. They elaborated that it is essential to “make sure that every woman has the same access to the technology” (HP3) and emphasized the potential discrimination against those who do not have access to GDM self-management technology.

### Phase-2 Results (Women With GDM Using the GDm-Health App)

#### Overview

In phase 2, a total of 10 women with GDM contributed by discussing their experiences of using a state-of-the-art, UK-based, GDM management app (GDm-Health). The purpose of this phase was to discover how well this app met the needs of women with GDM that were identified in phase 1. GDm-Health’s interface and functionalities have been briefly documented in Multimedia Appendix 6 [30,40,41].

The average age of women was 34.5 (SD 4.881) years, and the average period of gestation was 29 (SD 7.466) weeks (data for the gestational age of a woman were missing). All women (10/10, 100%) used smartphones on a daily basis for different tasks and activities (Multimedia Appendix 3).

A summary of themes and subthemes obtained from women’s perceptions in phase 2 is shown in Table 3. Full definitions of these themes are available in Multimedia Appendix 4.

**Table 3:** Summary of themes and subthemes obtained from women’s perceptions in phase 2.

Themes and subthemes			Key findings
**Enhancing the information and functionalities**			
	Addressing women’s basic needs	Quick and automatic entry of BG^a^ values Reducing in-person clinical consultations	
	Optimizing the data recording functionalities	Need for having specific space for logging different data Need for having the ability to edit the time of BG entry	
	Optimizing the communication functionalities	Need for having different ways to communicate with HPs^b^ Need for having a means for communication with other women with GDM^c^	
	Improving the information on the app	Insufficient information Need to have essential information such as recipes, safe exercise, and women’s stories	
**Optimizing the interface design**			
	Optimizing the data recording interface design	Not having personalized options Facing difficulty to record data via multiple screens	
	Optimizing the data visualizations	Difficult to differentiate between BG values on the scatterplot graph Need to consider different learning abilities Unclear layout for showing the availability of features or contents on the app	

^a^BG: blood glucose.

^b^HP: health professional.

^c^GDM: gestational diabetes mellitus.

#### Theme 1: Enhancing the Information and Functionalities

##### Addressing Women’s Basic Needs

Data recording features in the GDm-Health app were those that women found to be the most supportive of their basic GDM self-management needs. Of the 10 women, 2 (20%) mentioned their appreciation of the function that allows transferring BG readings automatically from their glucose meter to the app, which makes recording data quick and easy. However, 20% (2/10) of the women had a problem in syncing the app with their glucose meters, and woman 9 reported having an issue with sending a request call in the GDm-Health app:

...You’ve got to scan your phone onto the monitor and half the time, half the time my scanning onto the monitor doesn’t work.

Woman 1

Women valued the convenience of reducing in-person consultations by using GDm-Health. They appreciated that HPs would review their data once a week and were confident that they would contact “if there was any issue” (woman 2).

##### Optimizing the Data Recording Functionalities

Despite the ability to record data in GDm-Health meeting women’s needs, they believed that there were some restrictions in this feature. For example, women felt that the lack of functionalities for recording information such as activities and food were important limitations:

There’s not actually a section to record the food, so I’ve just been putting it in the comment section.

Woman 2

They found it “annoying” (women 4 and 5) to record all information except BG readings in the generic comment space.

Furthermore, participants indicated a need to edit the time of their BG test on the app, which is currently downloaded automatically from the BG meter to the app, similar to a time stamp, and it is not editable. This results in time discrepancies when the app is not synced with the BG meter at the time of testing:

I could have done my testing 2 hours ago, but it looks like I’m doing it at 5 o’clock when I did it at 3 o’clock.

Woman 9

##### Optimizing the Communication Functionalities

Although a feature to request a callback from a HP is available on the app, women perceived significant limitations in communication with HPs through GDm-Health. Women emphasized the lack of 2-way communication and suggested having different ways for women to communicate with HPs, for example, through SMS text messages:

...With the current app we can all, we can only ask a phone, a phone call back, but we can’t make a text.

Woman 4

...There’s no way to speak back...

Woman 7

Furthermore, the lack of communication with other women with GDM was another limitation of GDm-Health that was raised. Some women were interested in communicating with other women with GDM via the app mainly to get emotional support and for “not feeling alone” (woman 4). Woman 7 had already joined a Facebook group from Gestational Diabetes UK, and although she perceives its benefits, she further explained that women need to search to find it and require a Facebook account to join the group. Therefore, it would be helpful and more convenient for women to have communication groups within an app.

##### Improving the Information on the App

Limited and insufficient information was a common aspect mentioned by women regarding the information section of GDm-Health. The app lacks information perceived to be essential such as “recipe ideas, safe exercise” (woman 4), medication, and other women’s experiences. Woman 9 also indicated a need for providing information about GDM for family and friends to help them understand the condition and how it affects women. They also emphasized the importance of others’ “experiences and support outside of just the facts” (woman 5) about the GDM condition and its management as “women might be feeling quite vulnerable” (woman 5) and believed this could provide reassurance:

...Having stories from other people, is really, might be really reassuring for somebody.

Woman 2

Women also expressed that it would be more supportive to access all information on the app, “rather than just sending you directly to the NHS (National Health Service) website” (woman 5) or searching the internet by themselves. Woman 2 also believed this would ensure that the information is evaluated by professionals.

#### Theme 2: Optimizing the Interface Design

##### Optimizing the Data Recording Interface Design

Overall, 20% (2/10) of the women with GDM expressed ease in recording their data with the current layout of GDm-Health. The perception was owing to their familiarity with the interface over time and its use of simple drop-down boxes. However, others perceived that the interface design could be optimized to address women’s needs. For example, women mentioned not having personalized options for recording data and difficulty in recording data via multiple screens:

It's quite a clunky process on the GDM app..., I normally do it after a few readings, like after a day. So, I then have to go back and forward on screens.

Woman 2

Lack of personalization was one of the factors that women found challenging. An option to record the whole day’s data in a single attempt at the end of the day or to log data in different formats were felt to be missing. Women also indicated the inability to customize the meal type drop-down list options based on the number of times that they do blood tests, with woman 10 explaining that “the options don’t always marry up with what your clinical team ask you for.”

Some women also found it difficult to record data through GDm-Health owing to its multiscreen layout. It was seen to be inconvenient and time consuming:

...So you can’t see it at the same time as what your meal type and things like that are, so it’s better seen all on the one screen.

Woman 9

##### Optimizing the Data Visualizations

Data visualization in GDm-Health was another concern that 60% (6/10) of the women raised during their discussions. Women appreciated visualizing data as a graph and having quick access to it via the app. Some found the scatterplot graph used in the app easy to understand with data presented in chronological order, distinguished with color coding. A few women also reported features that helped them to understand the graph, such as “the thresholds for low blood sugar and high blood sugar” (woman 3):

You’ve got an option in the corner to change that, so you can choose to have a look at just breakfast, just lunch, just your evening meal.

Woman 5

However, half of the women (5/10, 50%) found it difficult to differentiate between BG values on the scatterplot graph. Some women perceived that a line graph would be easier to understand than a scatterplot graph for identifying trends and patterns. Moreover, woman 9 emphasized that people have different learning abilities, such as people with dyslexia. Therefore, providing various types of graphs would be helpful for women with a wide scope of learning abilities.

Others found the format to be inconvenient for comparing BG readings for different days by scrolling up and down the list of BG readings:

...You’ve got to scroll down with the current app, which isn’t very helpful, it’s not easy to compare days right now.

Woman 9

In general, women thought that there were necessary improvements to the interface design of GDm-Health, particularly regarding layout and data visualization to support their self-management.

## Discussion

### Principal Findings

#### Overview

In phase 1, both women and HPs believed that the pervasiveness and convenience of technology could support both the quality of women’s lives and the quality of HPs’ work. They identified recording data, visualizing data, access to essential and adequate GDM management information, and ability to communicate with HPs and other women with GDM as primary needs of women with GDM. They also highlighted their concerns about data privacy and security, lack of sufficient information, information reliability issues, and interface design issues of existing technologies that need to be addressed. Finally, they emphasized the technology’s limitations, such as lack of emotional and physical support, reliability of technology, and equality issues, that cause resistance to technology adoption.

Similarly, in phase 2, despite finding that the GDm-Health app met some of their basic needs, women perceived the functionality and interface design of its features, such as recording data, visualizing data, communication, and information, to be suboptimal.

We have discussed the findings from our thematic analyses of both phases from 3 perspectives: importance of women’s emotional and personal needs, personalization of data presentation, and personalization of data recording.

#### Importance of Women’s Emotional and Personal Needs

Phase 1 underlined women’s and HPs’ perceptions about the needs of women with GDM from self-management technology, such as recording data (including BG, food, and activity), access to information, and communication with HPs and other women with GDM. HPs identified women’s needs from a primarily medical perspective, whereas women with GDM were able to discuss their emotional and personal needs that helped to identify extra requirements that needed to be addressed. For example, women discussed the feeling of being upset and scared when diagnosed with GDM, consistent with a previous study by Lydon et al [42].

Although women in our study were concerned about technology’s limitations regarding proximity and emotional support during web-based clinical appointments, they saw how it could support their psychological well-being by providing or enhancing social and health care support through different means of communication. Another example was the role of partners, family, and friends in managing GDM, which was identified in our interviews. This is consistent with previous studies showing the benefits of support from family and friends [43]. Technology could play a key role by providing the materials and information for partners or families to enable them to enhance their support for women with GDM.

Furthermore, in phase 1, although HPs valued the communication with women with GDM, only our women interviewees indicated the need to have different ways of communicating with HPs. Similarly, in phase 2, women underlined the lack of different means of communication with HPs via the GDm-Health app as a primary issue. They thought that the availability of various communications, such as messaging HPs in non-urgent situations, would be helpful.

In phase 1, both women with GDM and HPs discussed the potential benefits of sharing experiences with other women and hearing their stories via a GDM self-management system. This was corroborated in phase 2, wherein women with GDM indicated the lack of such a forum as a limitation of GDm-Health. They believed that experiences from other women with GDM would support them emotionally in managing their condition. This is also evident in previous studies using GDM self-management systems [26,38,44], and in the studies by Leziak et al [45] and Yee et al [46] that explored the experiences of pregnant women with gestational or pregestational diabetes in using technology to support their diabetes conditions during pregnancy. In general, women in these studies wanted peer support to be provided via these systems [26,38,44,45]. They appreciated the communication with other women for exchanging stories and experiences via the GDM self-management technology to get emotional support [38,46] and empower them with a wide scope of knowledge to manage their condition [38,44,45]. However, none of these previous studies reported the potential benefits of women’s partners sharing their stories or experiences with other partners. In our study, women advocated for the support of their partners in helping them adhere to their new lifestyle, but it is less likely that they will be given information about how best to do so. Therefore, women’s partners might also need support, both to cope with the new circumstances and to help women in managing their GDM condition to reduce the potential complications for both women and their babies.

#### Personalization of Data Presentation

In phase 1 of our study, women with GDM and HPs believed that using technology would be helpful for GDM management. However, both groups underlined the importance of the layout of contents and user interface design of technology. They highlighted the necessary requirements of simplicity, user-friendliness, and improved data visualizations including a variety of charts and color coding. These improvements would support women to understand their data and optimize GDM management, which also could lead to self-empowerment in managing their condition. This is consistent with previous review findings that showed that improving data visualization would lead to enhancing the usability of GDM systems and empower women with GDM with self-awareness about their data [47].

In phase 2, although some women found the data visualizations on GDm-Health to be useful for GDM management, others found it difficult to compare BG readings for different days owing to the app’s “list” style presentation. In addition, most women (8/10, 80%) also found it difficult to understand GDm-Health’s scatterplot graph and suggested line graph or bar chart alternatives. Offering different chart types would enable women to choose the easiest one for them to understand their data for improving GDM self-management. Studies of previous prototype apps have included either line graphs or bar charts but do not discuss the logic behind using these specific visualizations [31,48]. Other previous studies have also identified lack of visualization clarity [49] or the need for help in interpreting data [20] as factors that obtain low satisfaction scores, further supporting the need for data visualization improvement.

#### Personalization of Data Recording and Information

Although the GDm-Health app met some of the needs outlined in phase 1 regarding recording data, most women (8/10, 80%) in phase 2 believed that it still required improvement in both functionality and interface design aspects. Women did not like to record all their non-BG data, such as food and activity, in a generic comment box and desired the ability to record these data in dedicated spaces. They also found it cumbersome to record their data via multiple screens and suggested that it would be easy to record the whole day’s data on a single screen. This is consistent with the study by Georgsson and Staggers [50], which revealed that users found it difficult and time consuming to record data in multiple steps in a diabetes mHealth system.

Personalization was also seen as important in terms of app-based information. In phase 1, both women with GDM and HPs valued access to information in different formats, such as video clips for people with various learning abilities. Women also emphasized the importance of trusted and clinically verified information. Similarly, in phase 2, women believed that the information section of GDm-Health provided limited and insufficient information and desired access to essential GDM information on the app itself rather than providing links to other websites and resources. These results are also evident in previous studies, where both women with GDM and HPs believed that information on similar GDM apps was insufficient and generic [31,33,35,38]. The need for having access to personalized information [33] and detailed information regarding GDM [31,35] also arose from these studies.

#### Summary

In general, women and HPs were interested in using technology for GDM management as supplementary care. The overall findings of both phase-1 and phase-2 analyses underlined similar points for improving the technology to optimize women’s GDM self-management. Improving the usability in terms of content layout, user interface design, and data visualization; providing a feature to record different data types; personalization; providing essential and adequate information for GDM management; and allowing various communication means with HPs and other women with GDM were common suggestions among all participants. Our study also highlighted the vital role of women’s involvement in identifying the needs and requirements for a GDM self-management system.

### Limitations

A strength of this study was the involvement of both women with GDM and HPs to obtain a wide scope of understanding from the main stakeholders of GDM technology. In addition, gathering women’s perceptions in 2 different periods while using different methods of GDM management before COVID-19 (using paper logbook) and after COVID-19 (using a smartphone app) provided a broad understanding. However, some women with GDM had limited time available owing to their physical and life restrictions, such that few opportunities were available to follow up on important points raised during interviews. Exclusion of non–English-speaking women may exclude their experience with health technology but does not exclude ethnic variation in the study population.

### Conclusions

Our analysis of interviews with women with GDM and HPs showed how both groups were interested in using GDM self-management technology. Both HPs and women with GDM identified the needs regarding GDM self-management, with the latter describing their emotional and personal needs and those related to clinical well-being. In revealing the importance of the role that women can play in developing the requirements of the GDM self-management system, we call for further studies that directly involve women with GDM in the design and development process.

## Appendix

Multimedia Appendix 1 Interview question guides for women with gestational diabetes mellitus and women in the postpartum period who have had gestational diabetes mellitus for phases 1 and 2.

Multimedia Appendix 2 Interview question guide for health care professionals for phase 1.

Multimedia Appendix 3 Demographic information about women.

Multimedia Appendix 4 Definition of the themes.

Multimedia Appendix 5 Demographic information about health care professionals.

Multimedia Appendix 6 GDm-Health app interface and functionalities.

## Abbreviations

BG: blood glucose
GDM: gestational diabetes mellitus
HP: health professional
mHealth: mobile health
NHS: National Health Service
