# Supplementary material for: Baseline Perceptions of Women With Gestational Diabetes Mellitus and Health Care Professionals About Digital Gestational Diabetes Mellitus Self-Management Health Care Technologies: Interview Study Among Patients and Health Care Professionals
Source: JMIR Hum Factors. 2023 Dec 19;10:e51691. doi: 10.2196/51691 (PMC10762626; doi:10.2196/51691)
Supplement: Multimedia Appendix 1 [file humanfactors_v10i1e51691_app1.docx]

Multimedia Appendix 1: Interview question guides for women with GDM and postpartum who have had GDM for phases 1 & 2

# Phase 1

## 1) The GDM Condition

### - Experiences of having GDM.

1) What was your reaction when diagnosed with GDM?

2) What was your gestational week when you were diagnosed with GDM?

3) Can you tell me about some of your pregnancy experiences having GDM? How have you coped with GDM so far?

### - Ways of managing the condition.

4) How do/did you manage your GDM condition? Or what do/did you use for managing your condition?

5) In your opinion what is the problem with using a log book for recording your data? What problem do you face when you are using a logbook?

6) What is the problem with the current care? Do you think technology can help with regard to these problems?

7) What do you think is the best way of managing your condition, and why?

## 2) Technology

### - Opinions about using technology regarding GDM management.

1) What is your opinion about using technology like a mobile app or a website to manage your GDM condition?

2) If you were to use a mobile app or a website to manage your GDM, would you be confident to use it? And would it be convenient? Could you explain why you feel that way?

3) Compare using technology with using a logbook - for example, differences in recording your blood glucose reading, your diet or physical activity. Which one do you prefer? Which one is easier to use, for example, for recording your data: a mobile app or a website or a logbook and please explain why you think this.

4) Do you use any mobile apps or any website to help manage your GDM condition? If yes, which one do you use and how do you find it? What do you like about it and what do you dislike about it?

5) If you have not used any technology, what is your opinion of using a mobile app or a website to help with your condition? Which would you prefer?

6) What help would you like to get from technology? What do you need from technology to help you for managing your GDM condition?

7) Do you think technology like a mobile app, or a website would be reliable to use? Do you trust technology to use? Why/why not? Can you expand your answer?

8) If you use a mobile app would you worry about the privacy of your health data? What do you think about storing your data in a mobile or laptop and transferring it to NHS servers with regard to privacy?

9) What would be the possible problems of using technology like a mobile or a website?

10) What is the benefit or advantages of using technology like a mobile app to manage your condition?

11) In your opinion, what would encourage women with GDM or you to use technology to manage their condition?

12) Which device or platform is more appropriate or convenient to use?

### - Perceptions about remote monitoring and safety concerns (confidence and comfort with receiving care remotely compared to clinical visits).

1) What are your perceptions about remote monitoring such as virtual visits or sharing data with healthcare professionals compared to clinical visits?

2) Would you like to have virtual visits (like Skype or some other social media using mobile or a laptop) rather than having clinical visits? Why? What would be possible problems or benefits of having virtual visits?

3) Would you like clinical visits to be replaced by these virtual visits, or just have reduced clinical visits and use virtual visits to complement these? Which one do you prefer and why?

4) In general, do you have any concerns about using technology like mobile apps?

# Phase 2

1) What do you think about the GDm-Health app that you using?

2) What do you like or dislike about it?

3) What do you think about the logging data and the graph features?

4) What do you think about the communication feature?

5) What do you think about the information provided on the GDm-Health app?
