# Supplementary material for: Baseline Perceptions of Women With Gestational Diabetes Mellitus and Health Care Professionals About Digital Gestational Diabetes Mellitus Self-Management Health Care Technologies: Interview Study Among Patients and Health Care Professionals
Source: JMIR Hum Factors. 2023 Dec 19;10:e51691. doi: 10.2196/51691 (PMC10762626; doi:10.2196/51691)
Supplement: Multimedia Appendix 2 [file humanfactors_v10i1e51691_app2.docx]

Multimedia Appendix 2: Interview question guide for healthcare professionals for phase 1

# A) Current care in Tayside for women with GDM

## Consultant

1. Could you please explain the current care practice for women with GDM?

- In your opinion, is there any advantage or disadvantage with the current care practice?
- How can we improve the current practice?
- What issues are important to you with regard to GDM self-management? Which aspects of GDM self-management are important in order to monitor or control the GDM condition?
- What criteria do you use to diagnose GDM in Scotland?
- Do you know, what is the prevalence of GDM in Tayside or in Scotland?

2) Is any technology being used currently with regard to GDM self-management? If yes, please provide a brief description of it?

## Diabetes Nurse

1. Could you please explain briefly the current care practice for women with GDM?
2. The current method is using a logbook or a paper written document for GDM self-management. Am I right?

- Do you think using a logbook is enough?
- In your opinion, is there any advantage and disadvantage with the current care practice?
- How we can improve it? Do you think technology such as a mobile app or a website could help?

## Dietician

1) What support do you provide for women with GDM regarding their diet? How do women with GDM prefer to record their diet?

- In your opinion, is there any advantage and disadvantage with the current method of recording?

2) How is diet information provided to women in the early stage of their diagnosis?

- Do you think it is difficult for women to remember all the information in details later on?
- Do you think technology could help? And how?

3) Do you offer any website or any mobile app to women for helping them with their diet? If the answer is yes, please provide a brief description of it?

## For ALL

1) Why do/don’t you offer any technology like a mobile app or a website to women to manage their condition?

2) Is there any additional support offered between clinic appointments? If not, do you think technology can provide help?

# B) HPs’ opinions about using technology with regard to GDM self-management and how technology can help

1) What are your thoughts about using technology such as a mobile app or a website for managing women with GDM? Why?

2) What do you like or dislike about using technology?

3) Do you think using a mobile app or a website would lead to better care or optimize the blood glucose control for women compared to current care (for example, access to women’s data in real time and providing feedback to patients in real time compared to seeing data at a next visit)?

4) In your opinion, can technology like a mobile app or website offer a useful and practical remote control for women who live far from the clinic?

5) What is your opinion about virtual visits such as via Skype compared to face-to-face clinical visits, with the fact that some of women with GDM need to come from rural area to attend their clinical appointments?

6) Do you think using a mobile app or a website should be considered as complementary care in addition to the current care system, or can it stand alone for managing women with GDM? Why?

# C) Barriers and facilitators of using technology

1) Considering current busy clinics, what would be more convenient (easier and time-saving method) for healthcare professionals to use during the clinical visits, technology such as a mobile app or a logbook? Why?

2) In your opinion, are these technologies reliable or can you trust them with regard to the accuracy of the data or their content? Why?

3) In your opinion, what do women need from a digital GDM system? What are the most important elements of self-management that women can get help from technology like a mobile app or a website?

4) As a healthcare professional, what do you want from a digital GDM self-management system for managing GDM? How could technology like a mobile app help you to manage or monitor women with GDM? What information do you need from a digital GDM system?

5) In general, what would be the problems or barriers of using technology like a mobile app by healthcare professionals?

6) What are the concerns of healthcare professionals with regard to technology?

7) What are the benefits of using technology for both women and HPs?
