# Supplementary material for: Baseline Perceptions of Women With Gestational Diabetes Mellitus and Health Care Professionals About Digital Gestational Diabetes Mellitus Self-Management Health Care Technologies: Interview Study Among Patients and Health Care Professionals
Source: JMIR Hum Factors. 2023 Dec 19;10:e51691. doi: 10.2196/51691 (PMC10762626; doi:10.2196/51691)
Supplement: Multimedia Appendix 3 [file humanfactors_v10i1e51691_app3.docx]

Multimedia Appendix 3: Demographic Information for women

# Phase 1

## Section A: Personal Information

| Women Demographic information | | | |
| --- | --- | --- | --- |
| Age (years) | Gestational weeks | Qualification | Occupation |
| 34 | 34 | PGDE | Teacher |
| 32 | 32 | HND-Equine Science | Not employed |
| 38 | Missing | A level | Customer service manager |
| 30 | 24 | Postgraduate | Dance teacher |
| 30 | 37 | MSc | Teacher |
| 30 | 29 | BA HONS | Civil servant |
| 39 | 28 | MBA | Academic |
| 27 | Postpartum | BSc | Clinical physiologist |
| 23 | 34 | Level 6 | 999 operator |

## Section B: Frequency of Use of Technology

How often do you use the following technology? Select the most appropriate for each device type.

|  | Daily | Several times per week | Several times per month | Several times per year | Never |
| --- | --- | --- | --- | --- | --- |
| Smartphone | 9/9 (100%) |  |  |  |  |
| Tablet Device | 3/9 (33%) | 1/9 (11%) | 2/9 (22%) | 1/9 (11%) | 2/9 (22%) |
| Laptop | 1/9 (11%) | 5/9 (55%) | 2/9 (22%) | 0 | 1/9 (11%) |
| Smartwatch | 1/9 (11%) | 1/9 (11%) | 0 | 1/9 (11%) | 6/9 (66%) |

## Section C: Tasks Completed using Technology

What tasks do you complete on your devices? Select all appropriate answers.

|  | Banking & Finance | Social Media | Shopping | Browsing | Watching a movie | Listening to music | Reading | Tracking your health |
| --- | --- | --- | --- | --- | --- | --- | --- | --- |
| Smartphone | 6/8 (75%) | 7/8 (87%) | 6/8 (75%) | 7/8 (87%) | 3/8 (37%) | 5/8 (62%) | 4/8 (50%) | 5/7 (71%) |
| Tablet | 2/8 (25%) | 2/8 (25%) | 3/8 (37%) | 2/8 (25%) | 7/8 (87%) | 4/8 (50%) | 5/8 (62%) | 1/7 (14%) |
| Laptop | 4/8 (50%) | 3/8 (37%) | 4/8 (50%) | 3/8 (37%) | 4/8 (50%) | 2/8 (25%) | 3/8 (37%) | 1/7 (14%) |
| Smartwatch | 0 | 0 | 0 | 0 | 0 | 1/7 (14%) | 0 | 3/8 (37%) |

## Section D: Situational use of Technology

Where do you use the following technology? Please select all appropriate tasks?

|  | Watching TV | With friends / family | Lying in bed | In the bathroom | Public Transport | Private Transport | Other |
| --- | --- | --- | --- | --- | --- | --- | --- |
| Smartphone | 2/8 (25%) | 5/8 (62%) | 5/8 (62%) | 5/8 (62%) | 7/8 (87%) | 5/8 (62%) | 1/7 (14%) |
| Tablet | 4/8 (50%) | 2/8 (25%) | 5/8 (62%) | 0 | 2/8 (25%) | 1/7 (14%) | 0 |
| Laptop | 0 | 1/7 (14%) | 3/8 (37%) | 0 | 2/8 (25%) | 0 | 4/8 (50%) |
| Smartwatch | 1/7 (14%) | 1/7 (14%) | 2/8 (25%) | 2/8 (25%) | 2/8 (25%) | 2/8 (25%) | 0 |

For question C and D: a woman did not answer question C and D.

# Phase 2

## Section A: Personal Information

| Women Demographic information | | | |
| --- | --- | --- | --- |
| Age (years) | Gestational weeks | Qualification | Occupation |
| 36 | 27 | HND | Receptionist |
| 28 | 28 | BSc Hons | Operations manager oil and gas company |
| 41 | 27 | BA(Hons) and PGCE | Teacher |
| 29 | Missing data | BA (Hons) | Store worker |
| 40 | 16 | CCT general surgery | Doctor |
| 36 | 36 | BSc Hons forensic psychobiology | Community staff nurse |
| 35 | 36 | Post graduate certificate | Deputy service manager |
| 27 | 32 | ADV Diploma in management accounting | Accountant |
| 37 | 29 | Postgraduate | Lawyer |
| 36 | 20 | BSc HONS and PGCE | Secondary teacher |

## Section B: Frequency of Use of Technology

How often do you use the following technology? Select the most appropriate for each device type.

|  | Daily | Several times per week | Several times per month | Several times per year | Never |
| --- | --- | --- | --- | --- | --- |
| Smartphone | 10/10 (100%) |  |  |  |  |
| Tablet Device | 0 | 0 | 5/10 (50%) | 2/10 (20%) | 3/10 (30%) |
| Laptop | 4/10 (40%) | 3/10 (3)5) | 1/10 (10%) | 1/10 (10%) | 1/10 (10%) |
| Smartwatch | 3/10 (30%) | 1/10 (10%) | 0 | 0 | 6/10 (60%) |

## Section C: Tasks Completed using Technology

What tasks do you complete on your devices? Select all appropriate answers.

|  | Banking & Finance | Social Media | Shopping | Browsing | Watching a movie | Listening to music | Reading | Tracking your health |
| --- | --- | --- | --- | --- | --- | --- | --- | --- |
| Smartphone | 3/10 (30%) | 4/10 (40%) | 4/10 (40%) | 2/10 (20%) | 0 | 0 | 0 | 1/10 (10%) |
| Tablet | 0 | 1/0 (10%) | 1/10 | 1/10 (10%) | 2/10 (20%) | 0 | 3/10 (30%) | 0 |
| Laptop | 1/10 (10%) | 3/10  (30%) | 3/10  (30%) | 2/10  (20%) | 0 | 0 | 1/10  (10%) | 0 |
| Smartwatch | 0 | 0 | 0 | 0 | 0 | 0 | 0 | 3/10 (30%) |

## Section D: Situational use of Technology

Where do you use the following technology? Please select all appropriate tasks?

|  | Watching TV | With friends / family | Lying in bed | In the bathroom | Public Transport | Private Transport | Other |
| --- | --- | --- | --- | --- | --- | --- | --- |
| Smartphone | 4/10 (40%) | 4/10 (40%) | 5/10 (50%) | 0 | 0 | 0 | 1/10 (10%) |
| Tablet | 2/10 (20%) | 2/10  (20%) | 1/10 (10%) | 1/10 (10%) | 0 | 0 | 2/10 (20%) |
| Laptop | 3/10 (30%) | 3/10 (30%) | 1/10 (10%) | 0 | 0 | 0 | 3/10 (30%) |
| Smartwatch | 0 | 0 | 0 | 0 | 0 | 0 | 4/10 (40%) |
