# Supplementary material for: Baseline Perceptions of Women With Gestational Diabetes Mellitus and Health Care Professionals About Digital Gestational Diabetes Mellitus Self-Management Health Care Technologies: Interview Study Among Patients and Health Care Professionals
Source: JMIR Hum Factors. 2023 Dec 19;10:e51691. doi: 10.2196/51691 (PMC10762626; doi:10.2196/51691)
Supplement: Multimedia Appendix 4 [file humanfactors_v10i1e51691_app4.docx]

Multimedia Appendix 4: Definition of the Themes

# Phase 1 women’s themes definitions

| Fitting with Women's  Lifestyle Constraints (Theme 1) | This theme included women’s discussion about their perceptions of the effect of using eHealth on their lifestyle with regard to GDM self-management. |
| --- | --- |
| Technology's Design not Meeting Women’s Need (Theme 2) | This theme included women’s discussion about their personal opinions of technology’s limitations regarding GDM management. This theme includes 3 sub-themes: need for wellbeing support, data integrity concerns, and inadequate information for women’s needs. |
| Optimizing Technology's Design to Meet Women's Need (Theme 3) | This theme included women’s discussion about their perceptions of how technology could support them and meet their needs to improve their self-management regarding their GDM condition. This theme includes 4 sub-themes: data recording options, empowerment through understanding, improving communication, and optimizing user interface design. |

# Phase 1 HPs’ themes definitions

| Optimizing Technology Design to Improve Quality of Care (Theme 1) | This theme included HPs’ perceptions about improving technology to help them manage women with GDM and support women’s well-being and their life quality. This theme includes 2 sub-themes: optimizing the efficiency of care and communication, and decreasing HPs’ workload and improving women’s wellbeing |
| --- | --- |
| Technology to Support Women’s Independence (Theme 2) | This theme included HPs’ perceptions about how to improve technology for helping women to manage their GDM condition and supporting women to become more independent regarding GDM management. This theme includes 3 sub-themes: helping women to understand their data, increasing women's knowledge and motivation, and user interface design. |
| Limitations in Care System and Facilities (Theme 3) | This theme included HPs perceptions about the limitation of using GDM management technologies in the care system and also general limitations of using technology that would cause resistance to fully accepting technology regarding GDM management. |

# Phase 2 women’s themes definitions

| Enhancing Information and Functionalities (Theme 1) | This theme included women’s perceptions about how the functions (from the GDm-Health app) meet their needs. They also expanded their discussion to highlight how to improve the functionality and information section of the app to support their needs. This theme includes 3 sub-themes: addressing women’s basic needs, optimizing recording data functionalities, optimizing communication functionalities, and improving information on the app. |
| --- | --- |
| Optimizing interface design (Theme 2) | This theme included women’s perceptions of the positive points of the GDm-Health app interface design and the existing limitations and inconveniences of its layout that need to be improved. This theme includes 2 sub-themes: optimizing recording data interface design, and optimizing data visualizations. |
