# Supplementary material for: Baseline Perceptions of Women With Gestational Diabetes Mellitus and Health Care Professionals About Digital Gestational Diabetes Mellitus Self-Management Health Care Technologies: Interview Study Among Patients and Health Care Professionals
Source: JMIR Hum Factors. 2023 Dec 19;10:e51691. doi: 10.2196/51691 (PMC10762626; doi:10.2196/51691)
Supplement: Multimedia Appendix 5 [file humanfactors_v10i1e51691_app5.docx]

Multimedia Appendix 5: Demographic Information for Healthcare professionals

# Section A: Personal Information

| Healthcare professionals (HPs) Demographic information | | | |
| --- | --- | --- | --- |
| Age | Gender | Qualification | Occupation |
| 38 | Female | BSc Dietetics | Diabetes specialist Dietitian |
| 54 | Female | BA | Diabetes specialist nurse |
| 30 | Female | MSc | Diabetes specialist Dietitian |
| 40 | Female | MRCO, PhD | Doctor |
| 48 | Female | Diploma | Midwife |
| 45 | Female | Postgraduate | Doctor |
| 31 | Female | BSc Nursing | Diabetes specialist nurse |

# Section B: Frequency of Use of Technology

How often do you use the following technology? Select the most appropriate for each device type.

|  | Daily | Several times per week | Several times per month | Several times per year | Never |
| --- | --- | --- | --- | --- | --- |
| Smartphone | 7/7 (100%) |  |  |  |  |
| Tablet Device | 3/7 (42%) | 2/7 (28%) | 2/7 (28%) |  |  |
| Laptop | 4/7 (57%) |  | 2/7 (28%) | 1/7 (14%) |  |
| Smartwatch | 2/7 (28%) |  |  | 5/7 (71%) |  |

# Section C: Tasks Completed using Technology

What tasks do you complete on your devices? Select all appropriate answers.

|  | Banking & Finance | Social Media | Shopping | Browsing | Watching a movie | Listening to music | Reading | Tracking your health |
| --- | --- | --- | --- | --- | --- | --- | --- | --- |
| Smartphone | 7/7 (100%) | 7/7 (100%) | 7/7 (100%) | 6/7 (85%) | 3/7 (42%) | 6/7 (85%) | 3/7 (42%) | 7/7 (100%) |
| Tablet | 3/7 (42%) | 3/7 (42%) | 5/7 (71%) | 4/7 (57%) | 5/7 (71%) | 2/7 (28%) | 6/7 (85%) | 2/7 (28%) |
| Laptop | 3/7 (42%) | 2/7 (28%) | 5/7 (71%) | 5/7 (71%) | 3/7 (42%) | 3/7 (42%) | 2/7 (28%) | 2/7 (28%) |
| Smartwatch | 0 | 0 | 0 | 0 | 0 | 0 | 0 | 2/7 (28%) |

# Section D: Situational use of Technology

Where do you use the following technology? Please select all appropriate tasks.

|  | Watching TV | With friends / family | Lying in bed | In the bathroom | Public Transport | Private Transport | Other |
| --- | --- | --- | --- | --- | --- | --- | --- |
| Smartphone | 7/7 (100%) | 6/7 (85%) | 6/7 (85%) | 5/7 (71%) | 4 (57%) | 6/7 (85%) | 1/7 (14%) |
| Tablet | 6/7 (85%) | 5/7 (71%) | 5/7 (71%) | 1/7 (14%) | 0 | 0 | 03/ |
| Laptop | 3/7 (42%) | 2/7 (28%) | 1/7 (14%) | 0 | 0 | 0 | 2/7 (28%) |
| Smartwatch | 1/7 (14%) | 0 | 0 | 0 | 2/7 (28%) | 2/7 (28%) | 0 |
