# Supplementary material for: Baseline Perceptions of Women With Gestational Diabetes Mellitus and Health Care Professionals About Digital Gestational Diabetes Mellitus Self-Management Health Care Technologies: Interview Study Among Patients and Health Care Professionals
Source: JMIR Hum Factors. 2023 Dec 19;10:e51691. doi: 10.2196/51691 (PMC10762626; doi:10.2196/51691)
Supplement: Multimedia Appendix 6 [file humanfactors_v10i1e51691_app6.pdf]

Multimedia Appendix 6: GDm-Health app interface and functionalities

The GDm-Health app was used as a part of a research pilot study in Oxford in 2014 [1] and was commercially launched by Sensyne Health in 2018 [2].

GDm-Health is a cross-platform smartphone app that helps women with GDM and their HPs to manage GDM. It allows women with GDM to record and track their blood glucose readings and input additional comments. This information can be immediately accessed by HPs through a corresponding desktop application.

The GDm-Health application includes 2 parts: 1) A patient-facing mobile app, which is downloaded to the patient's mobile device, and 2) A secure clinician-facing website designed to allow remote blood glucose monitoring.

In this appendix, the main interface and functionality of the patient's application are briefly described. The below images are screenshots from a short video clip available on the app's Google Play Store presence [3].

Reading data

The "My Reading" screen includes two parts: 1) A scatter plot graph, and 2) A daily view.

1) The scatter plot graph is located at the top of the "My Reading" page (see Image B). Color coding is used for data points (blue represents hypoglycemia, green represents normal BG values, and red represents hyperglycemia). The horizontal axis shows days, and the vertical axis shows BG values.

2) Under the scatter plot, the BG values are displayed based on day, time, and meal type. The most recent BG values are shown at the top under the graph. By scrolling down, previous BG values for different days can be accessed. The same color coding is used for showing BG values.

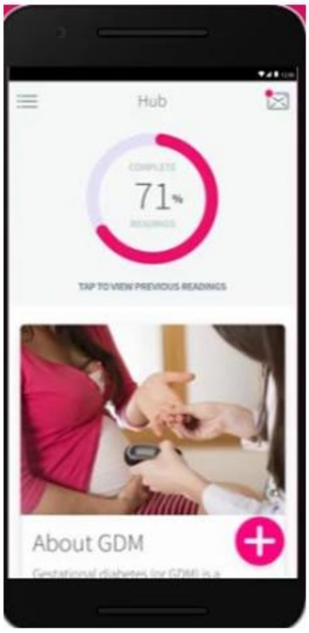

A) Hub screen

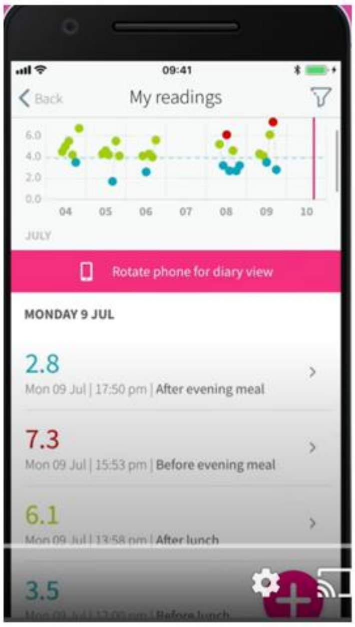

B) My readings screen (including graph and daily view)

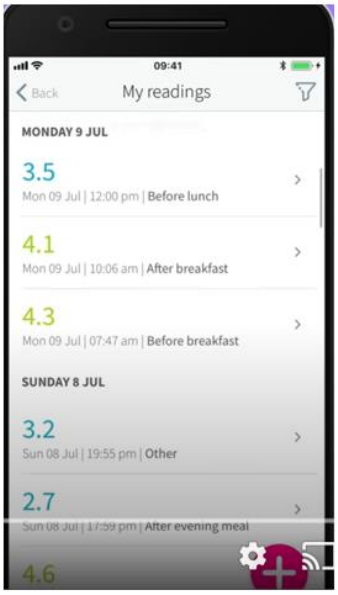

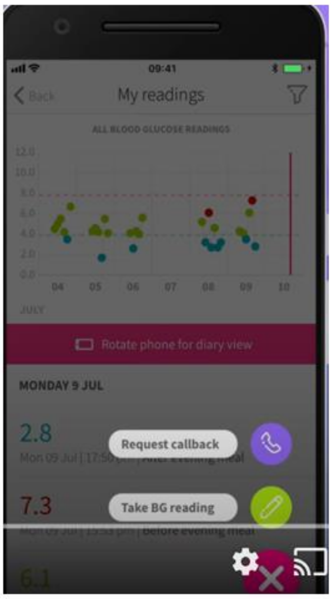

C) Daily view

D) Options for plus button

The 'plus' icon at the bottom-right of all pages provides two other options for women: "Request callback" and "Take BG reading" (see Image D).

When the mobile version of the app is rotated to a horizontal position, the "My Reading" page will show a diary view (Image E) on the screen with BG values in a tabular format. The first column includes dates, and the other six columns are organized based on the meal types: two columns for Breakfast (before and after), two columns for Lunch (before and after), and two columns for Dinner (before and after). A small square red file is available for some of the BG values that can be expanded to show women's comments.

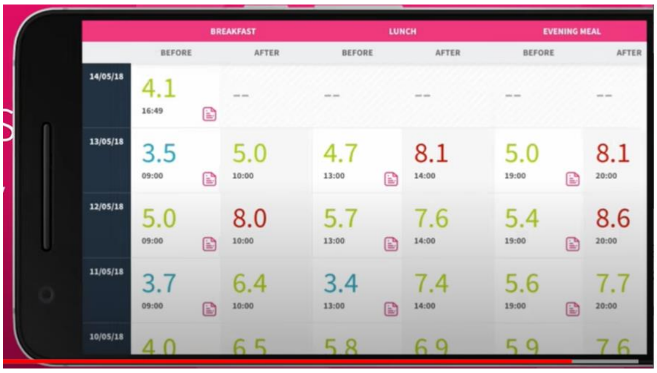

E) Diary view

Recording data

There are two ways to record BG reading values: 1) manually (using a free-text option), and 2) scanning (a BG reading will be transferred automatically from the patient's glucose meter to the app). The date and time are displayed on the BG recording screen. However, there is no function available for editing the time that the reading took place.

The recording data function is performed through multiple screens. After recording BG, the "Meal type" screen (Image H) allows patients to choose their meal type via a drop-down list. The next screen provides a field to record women's medication (free text option) (image I), followed by the "Comment" screen (free text option) (image J) to record women's comments or data such as food. In the Comment screen, there are two buttons for "Submit" and "Back" options. The meal type and medication screens have "Back" and "Next" buttons.

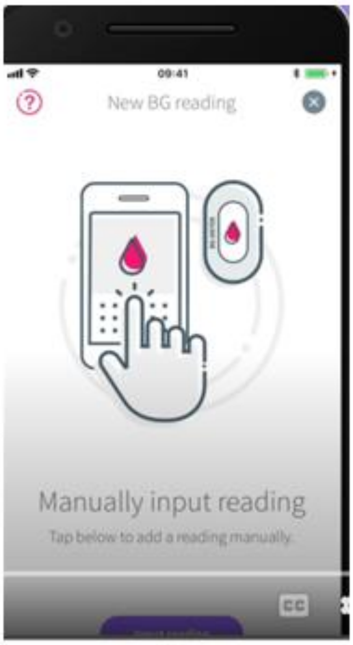

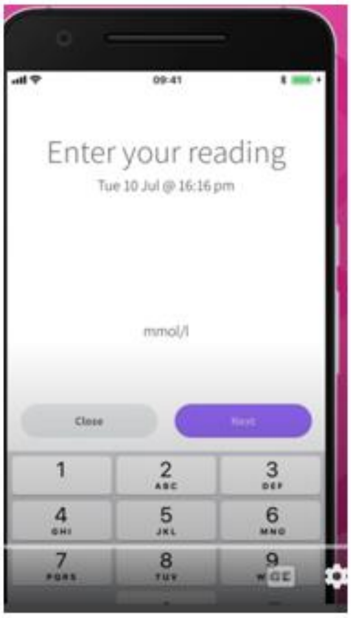

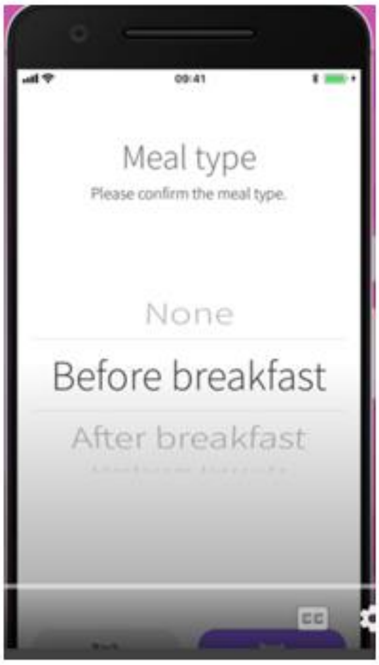

F) New BG reading screen

G) Recording BG reading's value

H) Choosing meal type

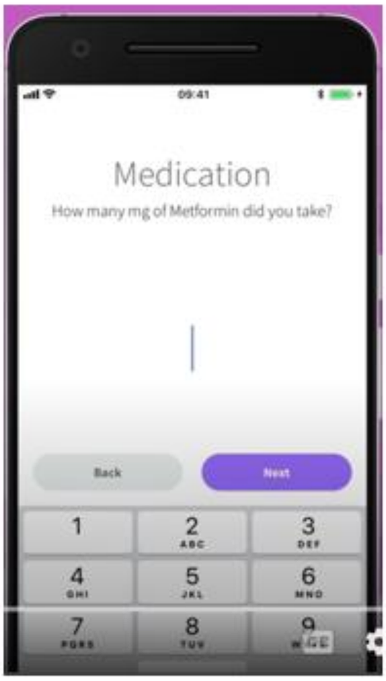

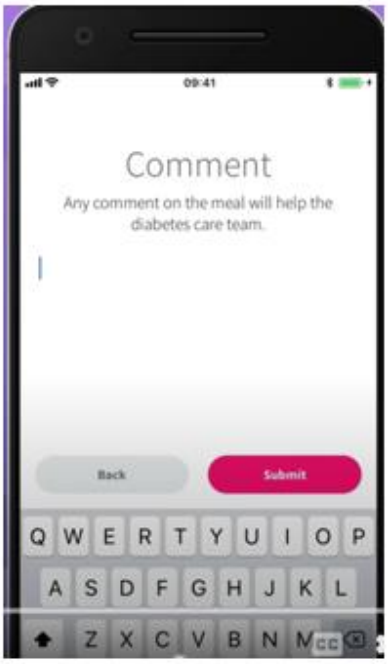

I) Recording medication

J) Recording any comments including food

References

[1] Mackillop L, Loerup L, Bartlett K, Farmer A, Gibson OJ, Hirst JE, Kenworthy Y, Kevat DA, Levy JC, Tarassenko L. Development of a real-time smartphone solution for the management of women with or at high risk of gestational diabetes. Journal of diabetes science and technology. 2014 Nov; 8(6):1105-14. PMID: 25004915 DOI: 10.1177/1932296814542271

[2] BBH, Building Better Health, Official launch of GDm-Health, 2018 https://www.buildingbetterhealthcare.com/news/article_page/Official_launch_of_GDm-Health/148302 [Accessed 29/06/2023]

[3] GDm-Health app, https://play.google.com/store/search?q=gdm-health+app&c=apps&hl=en&gl=US

[Accessed 08/10/2023]
